# Acupuncture for post-stroke recovery: a retrospective cohort study on motor function and quality of life

**DOI:** 10.3389/fneur.2026.1794260

**Published:** 2026-05-04

**Authors:** Min Su, Shiqi Cheng, Wei Yang, Xun Pan, Qiuju Su, Peng Yuan

**Affiliations:** 1 Department of Acupuncture, The Affiliated Wuxi People's Hospital of Nanjing Medical University, Wuxi People's Hospital, Wuxi Medical Center, Nanjing Medical University, Wuxi, China; 2 Department of Rehabilitation Medicine, The Affiliated Wuxi People's Hospital of Nanjing Medical University, Wuxi People's Hospital, Wuxi Medical Center, Nanjing Medical University, Wuxi, China

**Keywords:** acupuncture, electroacupuncture, Fugl-Meyer Assessment, motor function, quality of life, stroke rehabilitation

## Abstract

**Background:**

Stroke remains a leading cause of long-term disability worldwide. Acupuncture has been increasingly integrated into post-stroke rehabilitation programs, yet evidence regarding combined acupuncture protocols remains limited.

**Objective:**

To evaluate the effectiveness of combined acupuncture therapy (scalp acupuncture, electroacupuncture, and body acupuncture) plus conventional rehabilitation compared with conventional rehabilitation alone on motor function recovery and quality of life in patients with subacute stroke.

**Methods:**

This retrospective cohort study analyzed data from 108 patients with first-ever ischemic or hemorrhagic stroke admitted to our rehabilitation center between January 2022 and December 2024. Patients were divided into the acupuncture group (*n* = 54), receiving combined acupuncture plus conventional rehabilitation for 4 weeks, and the control group (*n* = 54), receiving conventional rehabilitation alone. Primary outcomes included Fugl-Meyer Assessment (FMA) for motor function and Stroke-Specific Quality of Life Scale (SS-QOL). Secondary outcomes included Modified Barthel Index (MBI), National Institutes of Health Stroke Scale (NIHSS), and modified Rankin Scale (mRS). Assessments were performed at baseline, post-treatment (4 weeks), and 12-week follow-up.

**Results:**

Baseline characteristics were comparable between groups (all *p* > 0.05). After 4 weeks of treatment, the acupuncture group demonstrated significantly greater improvement in FMA scores (61.28 ± 11.85 vs. 52.15 ± 10.42, *p* < 0.001) with a mean difference of 9.13 points (95% CI: 5.89–12.37). The acupuncture group also showed superior outcomes in MBI (73.58 ± 12.34 vs. 62.04 ± 11.56, *p* < 0.001), NIHSS (5.52 ± 1.89 vs. 7.62 ± 2.15, *p* < 0.001), and SS-QOL (181.35 ± 26.48 vs. 163.52 ± 24.86, *p* < 0.001). At 12-week follow-up, these improvements were sustained. Favorable functional outcome (mRS 0–2) was achieved in 79.6% of the acupuncture group versus 53.7% of controls (OR = 3.38, 95% CI: 1.52–7.51, *p* = 0.003). No serious adverse events occurred.

**Conclusion:**

Combined acupuncture therapy integrated with conventional rehabilitation was associated with significantly improved motor function and quality of life in patients with subacute stroke compared with conventional rehabilitation alone. These benefits persisted at 12-week follow-up. Confirmation through rigorous randomized controlled trials with sham acupuncture controls is warranted.

## 1 Introduction

Stroke remains the second leading cause of death and the third leading cause of disability-adjusted life years globally, imposing substantial burdens on patients, families, and healthcare systems (1). Despite advances in acute stroke management, approximately 50–70% of survivors experience persistent motor dysfunction affecting their independence and quality of life (2). Post-stroke motor recovery is a complex process involving neuroplasticity, functional reorganization, and compensatory mechanisms, necessitating comprehensive rehabilitation strategies (3).

Conventional rehabilitation approaches, including physical therapy and occupational therapy based on neurodevelopmental techniques, constitute the cornerstone of post-stroke recovery (4). However, the extent of functional recovery achieved through conventional methods alone remains limited, particularly for moderate-to-severe motor impairment (5). Consequently, there is growing interest in complementary therapies that may enhance neurological recovery when combined with standard rehabilitation protocols.

Acupuncture, a key component of Traditional Chinese Medicine (TCM), has been practiced for thousands of years in China for stroke treatment and has gained international recognition as a complementary therapy (6). The World Health Organization has recommended acupuncture as an adjunctive treatment for stroke sequelae, and it has been progressively endorsed in clinical guidelines of numerous countries (7). The therapeutic mechanisms of acupuncture in stroke rehabilitation are multifaceted, including promotion of neuroplasticity through brain-derived neurotrophic factor (BDNF) upregulation, modulation of inflammatory responses via the NF-κB pathway, enhancement of cerebral blood flow, and regulation of neurotransmitter release (8–10).

Recent evidence suggests that combined acupuncture protocols—integrating scalp acupuncture, electroacupuncture, and traditional body acupuncture—may provide superior therapeutic effects compared with single-modality treatments (11). Scalp acupuncture, based on the somatotopic organization of the motor cortex, directly stimulates cortical representation areas corresponding to impaired body regions (12). Electroacupuncture, with its standardized and quantifiable stimulation parameters, has demonstrated consistent effects on motor function recovery (13). Body acupuncture at specific acupoints along meridians complements these approaches by addressing systemic imbalances according to TCM theory (14).

The rationale for integrating these three acupuncture modalities is grounded in both theoretical complementarity and emerging empirical evidence. Each modality operates through distinct yet synergistic mechanisms targeting different levels of the neurorehabilitation cascade. Scalp acupuncture provides cortical-level neuromodulation by directly stimulating cortical representation areas corresponding to impaired body regions, thereby facilitating motor cortex reorganization and interhemispheric connectivity (12). Electroacupuncture delivers standardized peripheral neuromuscular stimulation with quantifiable parameters (frequency, intensity, waveform), which has been shown to activate both mu-opioid and delta-opioid receptor systems at alternating 2/100 Hz frequency, optimize neurotransmitter release, and promote axonal regeneration through BDNF upregulation (13, 15). Body acupuncture at specific meridian points addresses systemic regulatory imbalances according to TCM theory, modulating cerebral blood perfusion, autonomic nervous system balance, and inflammatory responses (14, 16). A systematic mapping review analyzing 3,645 randomized controlled trials identified that combined acupuncture protocols with rehabilitation training demonstrated the most robust evidence base among all acupuncture intervention patterns for post-stroke motor dysfunction (17). Similarly, Tian et al. (11) reported in a recent meta-analysis that scalp acupuncture combined with rehabilitation training yielded significantly greater improvements in motor function compared with either intervention alone. Thus, the multi-target, multi-pathway approach of combined acupuncture may create a more favorable neurobiological environment for motor recovery than any single modality.

However, despite the growing body of evidence from randomized controlled trials, real-world evidence from clinical practice settings evaluating combined acupuncture protocols remains scarce. Furthermore, comprehensive evaluation of both motor function and health-related quality of life (HRQOL) outcomes is essential for understanding the holistic impact of acupuncture therapy on stroke survivors.

The objective of this retrospective cohort study was to evaluate the effectiveness of combined acupuncture therapy (scalp acupuncture, electroacupuncture, and body acupuncture) integrated with conventional rehabilitation compared with conventional rehabilitation alone on motor function recovery and quality of life in patients with subacute stroke.

## 2 Methods

### 2.1 Study design and setting

This retrospective cohort study was conducted at the Department of Rehabilitation Medicine, the Affiliated Wuxi People’s Hospital of Nanjing Medical University, China, a tertiary teaching hospital with a specialized stroke rehabilitation unit. The study analyzed medical records of stroke patients admitted between January 2022 and December 2024. The study protocol was approved by the Institutional Ethics Committee and conducted in accordance with the Declaration of Helsinki. Due to the retrospective nature of the study, the requirement for individual written informed consent was waived by the ethics committee. All patient data were anonymized and de-identified prior to analysis. This study followed the Strengthening the Reporting of Observational Studies in Epidemiology (STROBE) guidelines for cohort studies and the Standards for Reporting Interventions in Clinical Trials of Acupuncture (STRICTA) guidelines for acupuncture intervention reporting.

### 2.2 Participants

Patients were identified through electronic medical record review. Inclusion criteria were (1): age 40–75 years (2); first-ever stroke confirmed by computed tomography (CT) or magnetic resonance imaging (MRI), including ischemic stroke or intracerebral hemorrhage (3); stroke onset within 14–90 days (subacute phase) (4); National Institutes of Health Stroke Scale (NIHSS) score of 5–15 (moderate severity) (5); stable vital signs for ≥48 h (6); Mini-Mental State Examination (MMSE) score ≥24, indicating adequate cognitive function for rehabilitation participation; and (7) complete medical records with documented baseline and outcome assessments.

Exclusion criteria were (1): transient ischemic attack or subarachnoid hemorrhage (2); recurrent stroke (3); severe cardiac, hepatic, or renal dysfunction (4); active malignancy (5); coagulation disorders or anticoagulant therapy contraindicating acupuncture (6); local skin infections at acupuncture sites (7); presence of cardiac pacemaker (8); severe cognitive impairment or aphasia preventing cooperation (9); history of epilepsy; and (10) prior acupuncture treatment for current stroke episode.

### 2.3 Group assignment

Patients were assigned to study groups based on the rehabilitation protocol they received during hospitalization. The acupuncture group comprised patients who received combined acupuncture therapy plus conventional rehabilitation, while the control group comprised patients who received conventional rehabilitation alone. Treatment allocation was primarily determined by patient preference, physician recommendation, and insurance coverage.

### 2.4 Interventions

#### 2.4.1 Conventional rehabilitation (both groups)

All patients received standardized conventional rehabilitation therapy delivered by licensed physical therapists and occupational therapists, including (1): Bobath neurodevelopmental technique focusing on postural control and movement facilitation (2); proprioceptive neuromuscular facilitation (PNF) patterns for upper and lower extremities (3); range of motion exercises and stretching (4); balance and gait training (5); activities of daily living (ADL) training; and (6) functional electrical stimulation for targeted muscle groups when indicated. Rehabilitation sessions were conducted for 45–60 min, twice daily, 5 days per week for 4 weeks (total 40 sessions). All patients also received standard medical care including antiplatelet therapy, blood pressure management, glycemic control, and lipid-lowering agents as indicated.

#### 2.4.2 Combined acupuncture therapy (acupuncture group)

In addition to conventional rehabilitation, patients in the acupuncture group received combined acupuncture therapy administered by licensed acupuncturists with >5 years of clinical experience in stroke rehabilitation. The acupuncture protocol was standardized according to published guidelines and consisted of three components:

##### 2.4.2.1 Scalp acupuncture

According to Jiao’s scalp acupuncture system, the motor area (MS6) corresponding to the affected limbs was selected on the contralateral scalp. For upper limb dysfunction, the middle-lower 2/5 of the motor area was needled; for lower limb dysfunction, the upper 1/5 of the motor area was needled. Sterile disposable stainless steel needles (0.30 mm × 40 mm, Huatuo Brand, Suzhou, China) were inserted at a 15–30° angle to the scalp surface and advanced subcutaneously for 30–35 mm. Rapid rotation manipulation (≥200 rotations/min) was performed for 1–2 min, then intermittently every 10 min during the 30-min retention period.

##### 2.4.2.2 Electroacupuncture

The following bilateral acupoints on the affected side were selected based on TCM meridian theory and modern neuroanatomical understanding: Jianyu (LI15), Quchi (LI11), Shousanli (LI10), Hegu (LI4), and Waiguan (SJ5) for upper extremity; Zusanli (ST36), Yanglingquan (GB34), Sanyinjiao (SP6), Jiexi (ST41), and Taichong (LR3) for lower extremity. Sterile disposable stainless steel needles (0.30 mm × 50 mm) were inserted perpendicularly to appropriate depths (15–35 mm depending on anatomical location). After achieving “Deqi” sensation (soreness, numbness, distension, or heaviness), electroacupuncture stimulation was applied using an SDZ-II electronic acupuncture apparatus (Hwato, Suzhou, China). Continuous wave at 2/100 Hz (alternating frequency) was delivered at an intensity tolerable to each patient (typically 1–3 mA) for 30 min.

##### 2.4.2.3 Body acupuncture

A semi-standardized protocol was employed, combining fixed core acupoints with individualized supplementary points based on TCM syndrome differentiation. Core acupoints administered to all patients included Baihui (GV20) and Sishencong (EX-HN1) for cognitive support and overall neural regulation. Supplementary acupoints were selected according to the patient’s predominant TCM syndrome pattern as determined by the treating acupuncturist at baseline (1): Qi deficiency with blood stasis syndrome (*n* = 23, 42.6%): Neiguan (PC6), Sanyinjiao (SP6), and Xuehai (SP10) to invigorate qi and resolve stasis (2); Liver yang hyperactivity syndrome (*n* = 16, 29.6%): Fengchi (GB20) and Taichong (LR3) to subdue liver yang and extinguish wind (3); Phlegm-dampness obstructing collaterals syndrome (*n* = 10, 18.5%): Fenglong (ST40) and Yinlingquan (SP9) to resolve phlegm and unblock collaterals (4); Wind-phlegm blocking collaterals syndrome (*n* = 5, 9.3%): Fengchi (GB20), Fenglong (ST40), and Hegu (LI4) to expel wind and dissolve phlegm. Standard needle manipulation (lifting-thrusting and rotating) was performed to achieve “Deqi,” followed by 30-min needle retention. This semi-standardized approach reflects contemporary clinical practice guidelines while ensuring reproducibility of the core protocol and allowing individualized treatment according to TCM principles.

Acupuncture treatment was administered once daily, 5 days per week, for 4 weeks (total 20 sessions). All acupuncture sessions were documented in detail, including acupoints, needle specifications, stimulation parameters, and patient responses.

### 2.5 Outcome measures

#### 2.5.1 Primary outcomes

##### 2.5.1.1 Fugl-Meyer Assessment (FMA)

The FMA motor subscale was used to evaluate motor function recovery. The scale comprises upper extremity (66 points) and lower extremity (34 points) components, with a total motor score of 100 points. Higher scores indicate better motor function. The FMA demonstrates excellent reliability (ICC = 0.95–0.98) and is the most widely used measure for motor function assessment in stroke rehabilitation research (18).

##### 2.5.1.2 Stroke-Specific Quality of Life Scale (SS-QOL)

The validated Chinese version of SS-QOL was administered to assess HRQOL. The SS-QOL comprises 49 items across 12 domains: mobility, energy, upper extremity function, work/productivity, mood, self-care, social roles, family roles, vision, language, thinking, and personality. Total scores range from 49 to 245, with higher scores indicating better quality of life (19).

#### 2.5.2 Secondary outcomes

##### 2.5.2.1 Modified Barthel Index (MBI)

The MBI was used to evaluate independence in ADL, covering 10 domains including feeding, bathing, grooming, dressing, bowel and bladder control, toilet use, transfers, mobility, and stairs. Scores range from 0 to 100, with higher scores indicating greater independence (20).

##### 2.5.2.2 National Institutes of Health Stroke Scale (NIHSS)

The NIHSS was used to assess neurological deficit severity. Scores range from 0 to 42, with higher scores indicating more severe neurological impairment (21).

##### 2.5.2.3 Modified Rankin Scale (mRS)

The mRS was used to evaluate global disability and functional outcome. Scores range from 0 (no symptoms) to 6 (death). Favorable functional outcome was defined as mRS 0–2 (functional independence) (22).

#### 2.5.3 Safety assessment

Adverse events were documented from medical records, including local reactions (hematoma, infection, pain), systemic reactions (dizziness, syncope), and any serious adverse events.

### 2.6 Data collection

Outcome assessments were performed at three time points: baseline (within 3 days of treatment initiation), post-treatment (4 weeks), and follow-up (12 weeks after treatment completion). All assessments were conducted by trained rehabilitation physicians who were blinded to treatment group allocation and were not involved in treatment delivery. To maintain blinding, assessment sessions were scheduled separately from treatment sessions, and assessors had no access to patients’ treatment records during the evaluation process. All assessors had undergone standardized training in FMA, NIHSS, MBI, and SS-QOL administration and demonstrated inter-rater reliability of ICC > 0.90 prior to study commencement. Baseline demographic and clinical characteristics extracted from medical records included age, sex, stroke type, stroke laterality, time from onset to treatment, comorbidities (hypertension, diabetes mellitus, hyperlipidemia, coronary heart disease, atrial fibrillation), and relevant laboratory findings.

### 2.7 Statistical analysis

Sample size calculation was based on detecting a clinically meaningful difference of 6 points in FMA scores (minimum clinically important difference) between groups, assuming a standard deviation of 12 points, α = 0.05, and power = 80%. The calculation indicated 51 patients per group; accounting for potential data incompleteness, a minimum of 54 patients per group was targeted.

Continuous variables were expressed as mean ± standard deviation (SD) and compared using independent samples *t*-tests for normally distributed data or Mann–Whitney U tests for non-normally distributed data. Normality was assessed using the Shapiro–Wilk test. Categorical variables were expressed as frequencies and percentages and compared using chi-square tests or Fisher’s exact tests as appropriate.

Within-group comparisons across time points were performed using repeated measures analysis of variance (ANOVA) with Bonferroni correction for multiple comparisons. Effect sizes were calculated as Cohen’s d for between-group differences, with values of 0.2, 0.5, and 0.8 representing small, medium, and large effects, respectively.

To address potential selection bias inherent to retrospective studies, propensity score matching (PSM) was performed as a sensitivity analysis. Propensity scores were estimated using logistic regression with covariates including age, sex, stroke type, time from onset, NIHSS baseline score, and comorbidities. One-to-one nearest-neighbor matching with a caliper of 0.2 was applied. Standardized mean differences (SMD) were calculated to assess covariate balance, with SMD < 0.1 indicating adequate balance.

Multivariate linear regression analysis was performed to identify independent predictors of motor function improvement, with FMA change score as the dependent variable and treatment group, age, sex, baseline NIHSS, time from onset, and comorbidities as independent variables.

Data completeness was ensured through inclusion criterion 7, which required complete medical records with documented baseline and outcome assessments at all three time points. All 108 enrolled patients had complete data for all primary and secondary outcomes, and no data imputation was required. As this is a retrospective cohort study rather than a randomized controlled trial, intention-to-treat analysis in its strict sense is not directly applicable. However, all enrolled patients completed the full treatment course and all scheduled assessments with no loss to follow-up or treatment discontinuation, resulting in a complete-case analysis with 100% retention that is functionally equivalent to a per-protocol analysis identical to intention-to-treat.

Statistical significance was set at *p* < 0.05 (two-tailed). All analyses were performed using SPSS version 26.0 (IBM Corporation, Armonk, NY, USA) and R version 4.2.1 (R Foundation for Statistical Computing, Vienna, Austria).

## 3 Results

### 3.1 Patient characteristics

**Figure 1:**
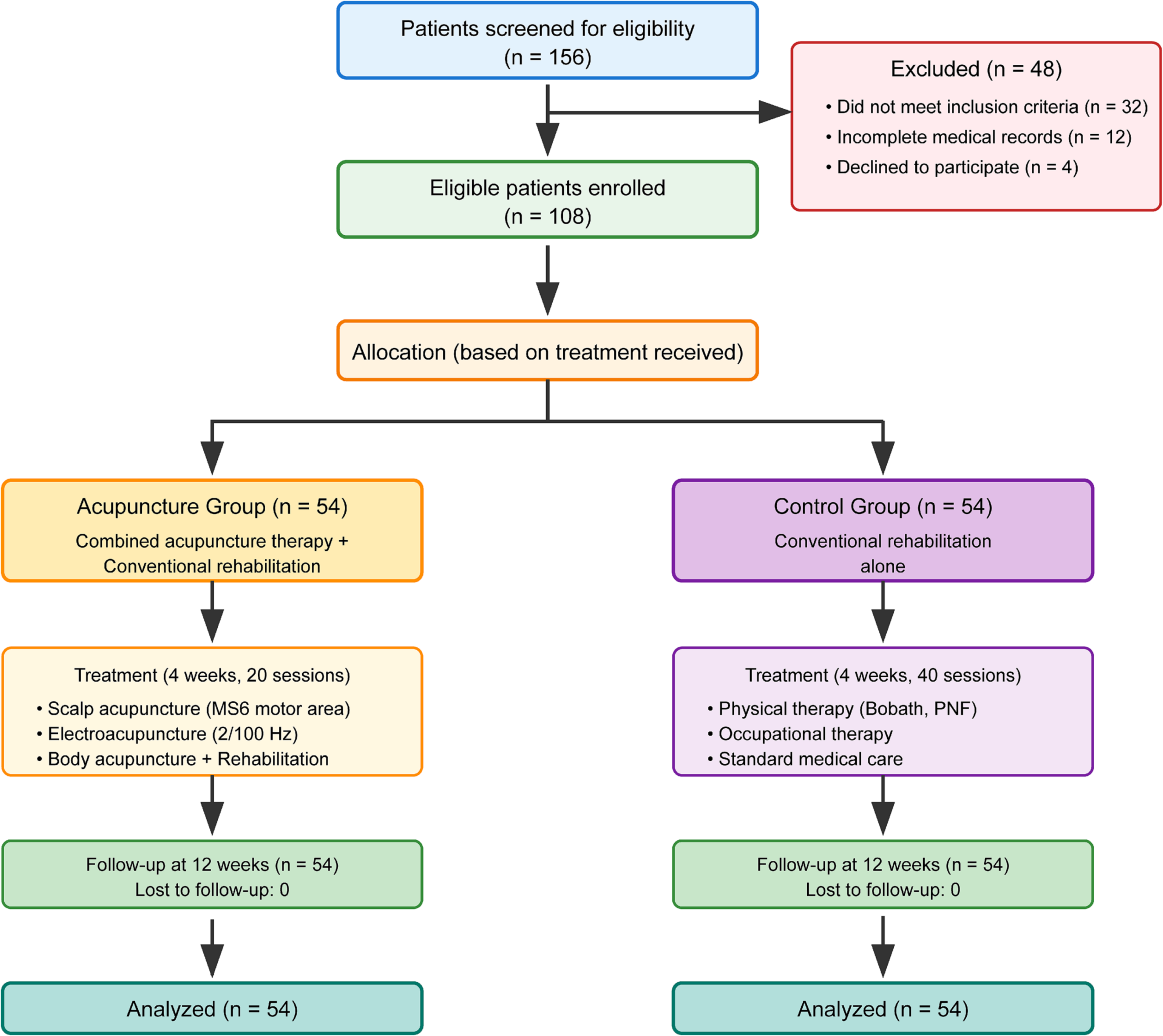
Patient flow diagram.

Between January 2022 and December 2024, 156 patients with stroke were screened for eligibility. After applying inclusion and exclusion criteria, 108 patients were included in the final analysis: 54 in the acupuncture group and 54 in the control group. The patient flow diagram is presented in Figure 1.

Baseline demographic and clinical characteristics of both groups are summarized in Table 1. The mean age was 62.58 ± 8.15 years in the acupuncture group and 63.85 ± 7.89 years in the control group (*p* = 0.412). Males comprised 63.0 and 61.1% of the acupuncture and control groups, respectively (*p* = 0.843). Ischemic stroke accounted for 87.0 and 85.2% of cases in the acupuncture and control groups, respectively (*p* = 0.782). The mean time from stroke onset to treatment initiation was 32.48 ± 12.76 days in the acupuncture group and 35.26 ± 14.08 days in the control group (*p* = 0.278). Prevalence of comorbidities, including hypertension (72.2% vs. 74.1%, *p* = 0.827), diabetes (31.5% vs. 33.3%, *p* = 0.839), was similar between groups.

**Table 1:** Baseline demographic and clinical characteristics.

Characteristic	Acupuncture group (*n* = 54)	Control group (*n* = 54)	*t*/*χ*^2^	*p* value
Demographics				
Age, years, mean ± SD	62.58 ± 8.15	63.85 ± 7.89	−0.823	0.412
Male, *n* (%)	34 (63.0)	33 (61.1)	0.039	0.843
Stroke characteristics				
Ischemic stroke, *n* (%)	47 (87.0)	46 (85.2)	0.077	0.782
Hemorrhagic stroke, *n* (%)	7 (13.0)	8 (14.8)	—	—
Left hemisphere affected, *n* (%)	28 (51.9)	27 (50.0)	0.037	0.847
Onset to treatment, days, mean ± SD	32.48 ± 12.76	35.26 ± 14.08	−1.089	0.278
Comorbidities, *n* (%)				
Hypertension	39 (72.2)	40 (74.1)	0.048	0.827
Diabetes mellitus	17 (31.5)	18 (33.3)	0.041	0.839
Hyperlipidemia	22 (40.7)	24 (44.4)	0.152	0.697
Coronary heart disease	8 (14.8)	9 (16.7)	0.071	0.79
Atrial fibrillation	6 (11.1)	7 (13.0)	0.088	0.767
Baseline outcome measures, mean ± SD				
FMA total score	42.65 ± 12.24	41.95 ± 11.87	0.303	0.762
FMA upper extremity	27.86 ± 8.52	27.35 ± 8.28	0.318	0.751
FMA lower extremity	14.79 ± 4.68	14.60 ± 4.52	0.216	0.829
NIHSS score	9.78 ± 2.38	10.12 ± 2.56	−0.721	0.472
MBI score	48.68 ± 14.16	47.42 ± 13.76	0.469	0.64
SS-QOL total score	142.86 ± 28.52	141.08 ± 27.35	0.338	0.736
mRS distribution, *n* (%)			0.929	0.628
mRS 2	14 (25.9)	12 (22.2)	—	—
mRS 3	29 (53.7)	31 (57.4)	—	—
mRS 4	11 (20.4)	11 (20.4)	—	—

SD, standard deviation; FMA, Fugl-Meyer Assessment; NIHSS, National Institutes of Health Stroke Scale; MBI, Modified Barthel Index; SS-QOL, Stroke-Specific Quality of Life Scale; mRS, modified Rankin Scale. Continuous variables compared using independent samples *t*-test; categorical variables compared using chi-square test.

Baseline outcome measures were comparable between groups: FMA (42.65 ± 12.24 vs. 41.95 ± 11.87, *p* = 0.762), NIHSS (9.78 ± 2.38 vs. 10.12 ± 2.56, *p* = 0.472), MBI (48.68 ± 14.16 vs. 47.42 ± 13.76, *p* = 0.640), and SS-QOL (142.86 ± 28.52 vs. 141.08 ± 27.35, *p* = 0.736). All baseline comparisons yielded *p* > 0.05, confirming adequate group comparability.

Treatment adherence was high and comparable between groups. The mean number of completed conventional rehabilitation sessions was 38.6 ± 1.8 out of 40 prescribed sessions (adherence rate: 96.5%) in the acupuncture group and 38.2 ± 2.1 (adherence rate: 95.5%) in the control group, with no significant between-group difference (*p* = 0.289). In the acupuncture group, the mean number of completed acupuncture sessions was 19.4 ± 0.9 out of 20 prescribed sessions (adherence rate: 97.0%). Reasons for missed sessions included temporary illness (e.g., upper respiratory infection), scheduling conflicts with diagnostic procedures, and personal reasons. No patient missed more than 3 rehabilitation sessions or 2 acupuncture sessions during the 4-week treatment period (Supplementary Table S2).

### 3.2 Primary outcomes

#### 3.2.1 Motor function (FMA)

At 4-week post-treatment assessment, the acupuncture group demonstrated significantly higher FMA scores compared with the control group (61.28 ± 11.85 vs. 52.15 ± 10.42, *p* < 0.001). The mean between-group difference was 9.13 points (95% CI: 5.89–12.37), representing a large effect size (Cohen’s d = 0.82). Within-group analysis revealed significant improvements from baseline in both groups (both *p* < 0.001), but the magnitude of change was significantly greater in the acupuncture group (18.63 ± 6.78 vs. 10.20 ± 5.86 points, *p* < 0.001).

**Figure 2:**
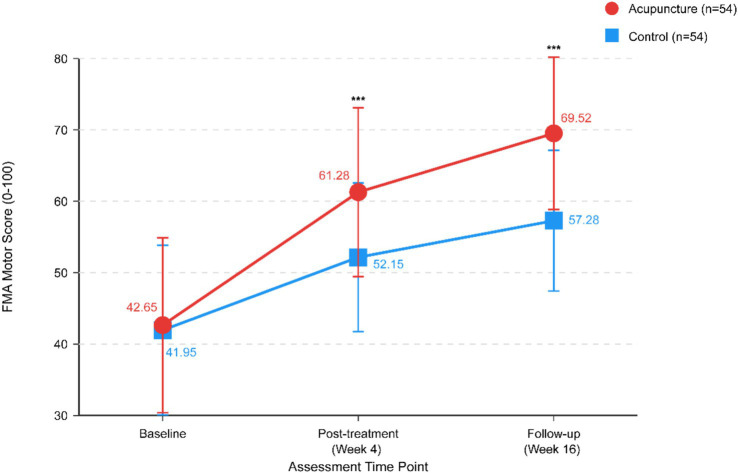
Fugl-Meyer Assessment (FMA) scores over time. Line graph depicting changes in FMA motor scores from baseline (Week 0) to post-treatment (Week 4) and follow-up (Week 16) for both treatment groups. Data presented as mean ± standard deviation. ***p < 0.001 for between-group comparison at each post-baseline time point.

At 12-week follow-up, the acupuncture group maintained significantly higher FMA scores (69.52 ± 10.68 vs. 57.28 ± 9.86, *p* < 0.001), with a mean difference of 12.24 points (95% CI: 8.31–16.17). Continued improvement from post-treatment to follow-up was observed in both groups, but the acupuncture group showed greater sustained recovery (Table 2; Figure 2).

**Table 2:** Comparison of outcome measures between groups at different time points.

Outcome	Group	Baseline	Post-tx (4wk)	Follow-up (12wk)	P(time)^a^
FMA total	Acupuncture	42.65 ± 12.24	61.28 ± 11.85***	69.52 ± 10.68***	<0.001
	Control	41.95 ± 11.87	52.15 ± 10.42***	57.28 ± 9.86***	<0.001
	P (between)	0.762	<0.001	<0.001	—
	Diff (95%CI)	—	9.13 (5.89–12.37)	12.24 (8.31–16.17)	—
FMA-UE	Acupuncture	27.86 ± 8.52	40.25 ± 8.18***	45.68 ± 7.52***	<0.001
	Control	27.35 ± 8.28	33.86 ± 7.56***	37.42 ± 7.18***	<0.001
	P (between)	0.751	<0.001	<0.001	—
FMA-LE	Acupuncture	14.79 ± 4.68	21.03 ± 4.25***	23.84 ± 3.86***	<0.001
	Control	14.60 ± 4.52	18.29 ± 3.92***	19.86 ± 3.68***	<0.001
	P (between)	0.829	<0.001	<0.001	—
NIHSS	Acupuncture	9.78 ± 2.38	5.52 ± 1.89***	4.02 ± 1.65***	<0.001
	Control	10.12 ± 2.56	7.62 ± 2.15***	6.82 ± 1.98***	<0.001
	P (between)	0.472	<0.001	<0.001	—
	Diff (95%CI)	—	−2.10 (−2.88,-1.32)	−2.80 (−3.50,-2.10)	—
MBI	Acupuncture	48.68 ± 14.16	73.58 ± 12.34***	84.12 ± 10.25***	<0.001
	Control	47.42 ± 13.76	62.04 ± 11.56***	68.28 ± 11.42***	<0.001
	P (between)	0.640	<0.001	<0.001	—
	Diff (95%CI)	—	11.54 (7.02–16.06)	15.84 (11.68–20.00)	—
SS-QOL	Acupuncture	142.86 ± 28.52	181.35 ± 26.48***	199.58 ± 24.85***	<0.001
	Control	141.08 ± 27.35	163.52 ± 24.86***	174.36 ± 23.42***	<0.001
	P (between)	0.736	<0.001	<0.001	—
	Diff (95%CI)	—	17.83 (8.02–27.64)	25.22 (16.02–34.42)	—

Data presented as mean ± SD. ^***^p < 0.001 vs baseline within the same group. FMA, Fugl-Meyer Assessment; NIHSS, National Institutes of Health Stroke Scale; MBI, Modified Barthel Index; SS-QOL, Stroke-Specific Quality of Life Scale; CI, confidence interval.

^a^*p* value for within-group comparison across time points (repeated measures ANOVA).

#### 3.2.2 Quality of life (SS-QOL)

The acupuncture group achieved significantly higher SS-QOL scores at 4-week post-treatment (181.35 ± 26.48 vs. 163.52 ± 24.86, *p* < 0.001), with a mean difference of 17.83 points (95% CI: 8.02–27.64). The improvement from baseline was 38.49 ± 15.18 points in the acupuncture group compared with 22.44 ± 12.76 points in the control group (*p* < 0.001).

**Figure 3:**
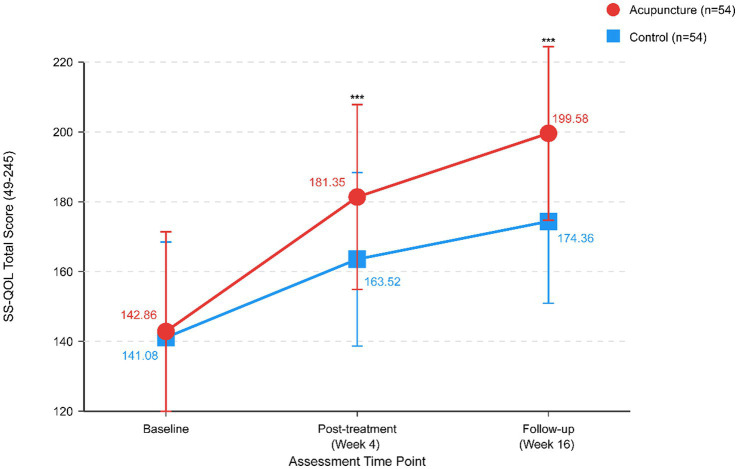
Stroke-Specific Quality of Life (SS-QOL) scores over time. Line graph illustrating changes in health-related quality of life measured by SS-QOL from baseline (Week 0) to post-treatment (Week 4) and follow-up (Week 16) for both treatment groups. Data presented as mean ± standard deviation. ***p < 0.001 for between-group comparison at each post-baseline time point.

At 12-week follow-up, SS-QOL scores remained significantly higher in the acupuncture group (199.58 ± 24.85 vs. 174.36 ± 23.42, *p* < 0.001), indicating sustained quality of life benefits (Figure 3).

### 3.3 Secondary outcomes

#### 3.3.1 Activities of daily living (MBI)

**Figure 4:**
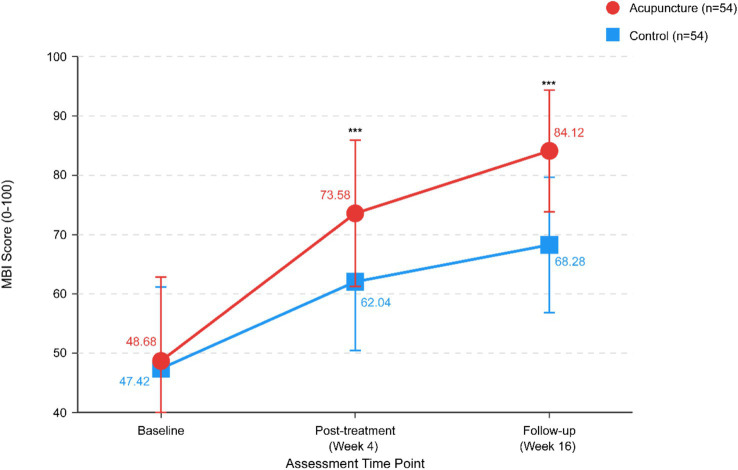
Modified Barthel Index (MBI) scores over time. Line graph showing changes in MBI scores (activities of daily living) from baseline (Week 0) to post-treatment (Week 4) and follow-up (Week 16) for both groups. Data presented as mean ± standard deviation. ***p < 0.001 for between-group comparison at each post-baseline time point.

The acupuncture group demonstrated significantly greater MBI scores at post-treatment (73.58 ± 12.34 vs. 62.04 ± 11.56, *p* < 0.001) and follow-up (84.12 ± 10.25 vs. 68.28 ± 11.42, *p* < 0.001). The between-group difference at post-treatment was 11.54 points (95% CI: 7.02–16.06), representing substantial clinical significance exceeding the minimum clinically important difference of 1.85 points (Figure 4).

#### 3.3.2 Neurological deficit (NIHSS)

NIHSS scores decreased significantly in both groups, indicating neurological improvement. At post-treatment, the acupuncture group showed significantly lower (better) NIHSS scores (5.52 ± 1.89 vs. 7.62 ± 2.15, *p* < 0.001). The reduction in NIHSS from baseline was 4.26 ± 1.78 points in the acupuncture group versus 2.50 ± 1.58 points in the control group (p < 0.001). At 12-week follow-up, the between-group difference persisted (4.02 ± 1.65 vs. 6.82 ± 1.98, p < 0.001).

#### 3.3.3 Functional outcome (mRS)

**Figure 5:**
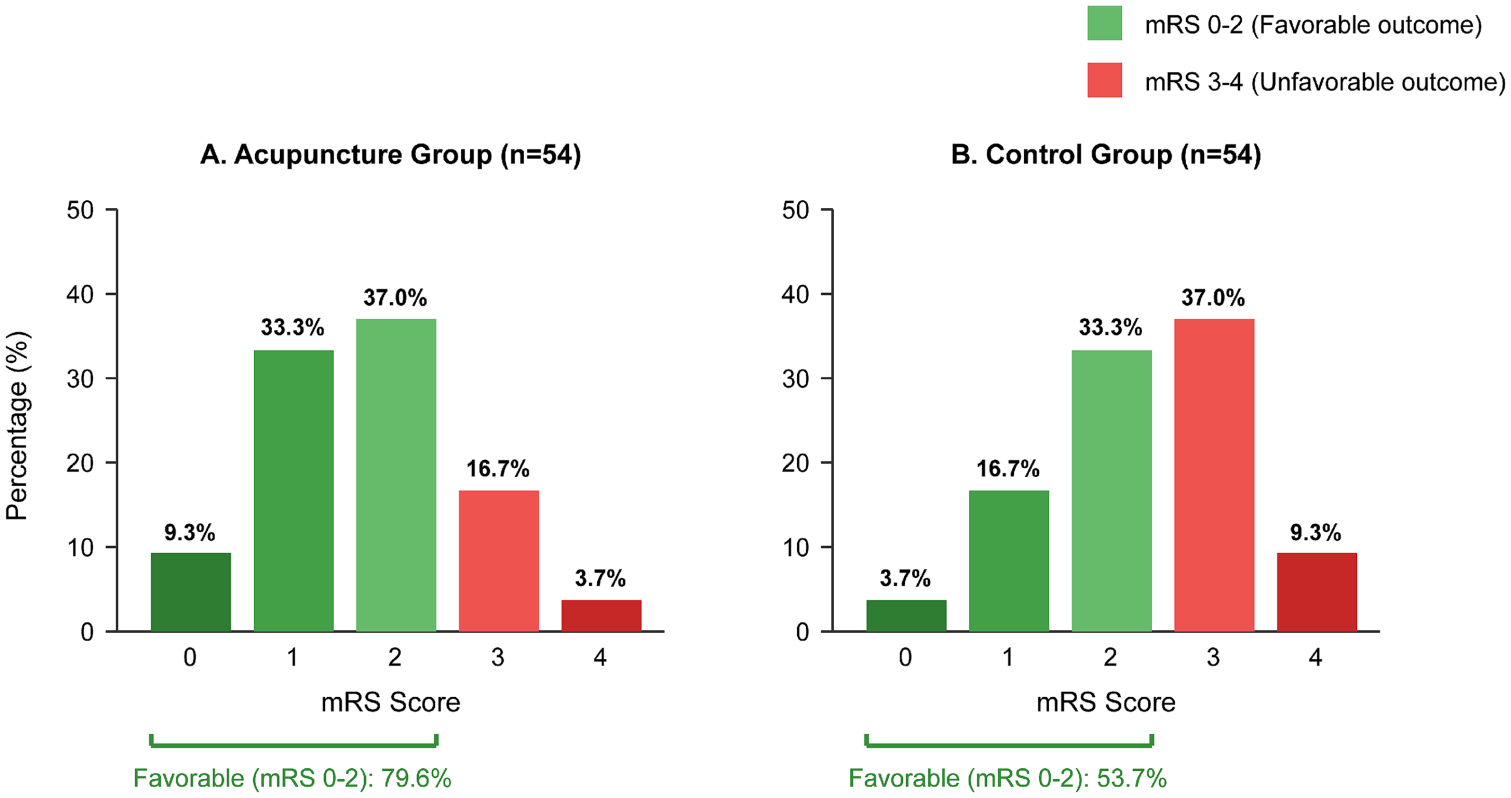
Modified Rankin Scale (mRS) distribution at post-treatment. Side-by-side bar charts comparing mRS score distributions between the acupuncture group (panel **A**) and control group (panel **B**) at post-treatment assessment.

At baseline, the distribution of mRS scores was similar between groups (*p* = 0.628). At post-treatment, favorable functional outcome (mRS 0–2) was achieved by 43 patients (79.6%) in the acupuncture group compared with 29 patients (53.7%) in the control group (*p* = 0.003). As illustrated in Figure 5, the odds ratio for favorable outcome was 3.38 (95% CI: 1.52–7.51), indicating that patients in the acupuncture group were more than three times as likely to achieve functional independence. At 12-week follow-up, the acupuncture group continued to show a significantly higher proportion of favorable outcomes (85.2% vs. 63.0%, *p* = 0.008; OR = 3.39, 95% CI: 1.38–8.33), confirming sustained functional benefits (Tables 3, 4; Figure 5).

**Table 3:** Modified Rankin Scale distribution and functional outcomes.

mRS Score	BL Acu	BL Ctrl	Post-tx Acu	Post-tx Ctrl	FU Acu	FU Ctrl
0, *n* (%)	0 (0.0)	0 (0.0)	5 (9.3)	2 (3.7)	8 (14.8)	3 (5.6)
1, *n* (%)	0 (0.0)	0 (0.0)	18 (33.3)	9 (16.7)	22 (40.7)	12 (22.2)
2, *n* (%)	14 (25.9)	12 (22.2)	20 (37.0)	18 (33.3)	16 (29.6)	19 (35.2)
3, *n* (%)	29 (53.7)	31 (57.4)	9 (16.7)	20 (37.0)	6 (11.1)	16 (29.6)
4, *n* (%)	11 (20.4)	11 (20.4)	2 (3.7)	5 (9.3)	2 (3.7)	4 (7.4)
Favorable (mRS 0–2), *n* (%)	14 (25.9)	12 (22.2)	43 (79.6)	29 (53.7)	46 (85.2)	34 (63.0)
*p* value	0.628		0.003		0.008	
OR (95% CI)	—		3.38 (1.52–7.51)		3.39 (1.38–8.33)	

BL, Baseline; Acu, Acupuncture Group; Ctrl, Control Group; Post-tx, Post-treatment (4 weeks); FU, Follow-up (12 weeks); mRS, modified Rankin Scale; OR, odds ratio; CI, confidence interval.

Favorable outcome defined as mRS 0–2 (functional independence). Chi-square test used for group comparisons.

**Table 4:** Multivariate linear regression analysis for predictors of FMA change score.

Variable	*β* coefficient	95% CI	*p* value
Treatment group (acupuncture vs. control)	7.86	5.42 to 10.30	<0.001
Age (per year increase)	−0.18	−0.32 to −0.04	0.012
Sex (male vs. female)	0.52	−1.86 to 2.90	0.665
Baseline NIHSS score (per point increase)	−0.85	−1.52 to −0.18	0.014
Time from onset (per day increase)	−0.12	−0.22 to −0.02	0.018
Stroke type (ischemic vs. hemorrhagic)	1.24	−1.58 to 4.06	0.385
Hypertension (yes vs. no)	−0.68	−3.24 to 1.88	0.598
Diabetes mellitus (yes vs. no)	−1.15	−3.62 to 1.32	0.358

Model summary: *R*^2^ = 0.458, Adjusted *R*^2^ = 0.426, *F* = 14.52, *p* < 0.001.

FMA, Fugl-Meyer Assessment; CI, confidence interval; NIHSS, National Institutes of Health Stroke Scale.

Dependent variable: FMA change score (post-treatment minus baseline).

### 3.4 Propensity score matching analysis

After 1:1 nearest-neighbor propensity score matching with a caliper of 0.2, 6 patients in each group could not be matched within the specified caliper distance due to differences in propensity score distributions at the tails, resulting in 48 matched pairs (48 patients per group) for the sensitivity analysis. Covariate balance was achieved with all SMD values <0.1 (Supplementary Table S1). In the matched cohort, the acupuncture group continued to demonstrate significant advantages in FMA (60.85 ± 11.52 vs. 51.68 ± 10.18, *p* < 0.001), MBI (72.96 ± 12.08 vs. 61.52 ± 11.24, *p* < 0.001), NIHSS (5.68 ± 1.92 vs. 7.78 ± 2.08, *p* < 0.001), and SS-QOL (180.52 ± 25.86 vs. 162.85 ± 24.12, *p* < 0.001) at post-treatment assessment. These results support the robustness of findings after accounting for measured confounders.

### 3.5 Multivariate regression analysis

Multivariate linear regression with FMA change score as the dependent variable identified treatment group (*β* = 7.86, 95% CI: 5.42–10.30, *p* < 0.001), younger age (*β* = −0.18, 95% CI: −0.32 to −0.04, *p* = 0.012), lower baseline NIHSS (*β* = −0.85, 95% CI: −1.52 to −0.18, *p* = 0.014), and shorter time from onset (*β* = −0.12, 95% CI: −0.22 to −0.02, *p* = 0.018) as independent predictors of greater motor function improvement. The model explained 42.6% of variance in FMA change scores (adjusted *R*^2^ = 0.426).

### 3.6 Safety

A total of 1,080 acupuncture sessions were administered to 54 patients. Minor adverse events were reported in 12 sessions (1.1%), including subcutaneous hematoma (*n* = 5), transient needle-site pain (*n* = 4), and mild dizziness (*n* = 3). All events resolved spontaneously without intervention. No serious adverse events, including infection, nerve injury, or pneumothorax, were observed. No patients withdrew from treatment due to adverse events (Table 5).

**Table 5:** Adverse events during acupuncture treatment.

Adverse event	Number of sessions (%)	Severity	Management	Outcome
Subcutaneous hematoma	5 (0.46)	Mild	None	Resolved spontaneously within 3–5 days
Needle-site pain	4 (0.37)	Mild	None	Resolved upon needle removal
Dizziness	3 (0.28)	Mild	Rest, supine position	Resolved within 5–10 min
Total	12 (1.11)	—	—	—
Serious adverse events	0 (0.00)	—	—	—

Total acupuncture sessions administered: 1,080 (54 patients × 20 sessions).

No patients withdrew from treatment due to adverse events.

## 4 Discussion

This retrospective cohort study provides real-world evidence suggesting that combined acupuncture therapy integrated with conventional rehabilitation is associated with significantly greater improvements in motor function recovery and quality of life in patients with subacute stroke compared with conventional rehabilitation alone. However, several important caveats must be acknowledged when interpreting these findings. First, the combined acupuncture protocol employed in this study is a multicomponent intervention comprising scalp acupuncture, electroacupuncture, and body acupuncture. The present study design does not allow isolation of the individual contributions of each component to the overall therapeutic effect. Future dismantling studies with factorial designs would be needed to determine the relative efficacy and potential synergistic effects of each modality. Second, as this is a retrospective, non-randomized study, the observed differences represent associations rather than causal relationships. Despite the use of propensity score matching and multivariate regression to address measured confounders, residual confounding from unmeasured variables cannot be excluded. These findings should therefore be considered hypothesis-generating and require confirmation through rigorous prospective randomized controlled trials.

With these caveats in mind, the acupuncture group showed a 9.13-point greater improvement in FMA scores, exceeding the established minimum clinically important difference of 4.25–7.25 points for motor function (23). These benefits extended to activities of daily living, neurological function, and global functional outcome, with advantages sustained at 12-week follow-up.

### 4.1 Comparison with previous studies

Our findings are consistent with recent meta-analyses and clinical trials evaluating acupuncture for post-stroke motor dysfunction. A systematic review by Ke et al. (17) analyzing 3,645 RCTs found that combined acupuncture protocols with rehabilitation training demonstrated superior efficacy compared with rehabilitation alone. The weighted mean difference in FMA reported in previous meta-analyses ranged from 8.5 to 12.8 points (24, 25), which aligns with our observed difference of 9.13 points.

The retrospective study by Hou et al. (26) examining electroacupuncture combined with herbal therapy in 126 stroke patients reported similar magnitudes of improvement in upper limb function. Similarly, Zhan et al. (27) demonstrated that scalp acupuncture therapy significantly improved motor function in a multi-center RCT, with changes in FMA scores consistent with our findings. The prospective cohort study by Jianfei et al. (28) using functional near-infrared spectroscopy confirmed that acupuncture combined with rehabilitation robot training improved both motor function and neural plasticity markers.

Our study extends previous findings by employing a comprehensive combined acupuncture protocol integrating scalp acupuncture, electroacupuncture, and body acupuncture—an approach identified as optimal in recent systematic mapping reviews (17). Furthermore, our inclusion of both motor function and quality of life outcomes provides a more holistic assessment of therapeutic benefits.

### 4.2 Mechanisms of action

The superior outcomes observed in the acupuncture group may be attributed to several neurobiological mechanisms. Scalp acupuncture directly stimulates cortical regions corresponding to motor function, potentially facilitating neuroplasticity and motor cortex reorganization (29). Neuroimaging studies have demonstrated that scalp acupuncture modulates fractional amplitude of low-frequency fluctuation (fALFF) in motor-related brain regions and enhances functional connectivity between hemispheres (30, 31), Specifically, interactive dynamic scalp acupuncture has been shown to increase interhemispheric connectivity in the supplementary motor area and primary motor cortex, providing a neuroanatomical basis for the observed motor function improvements (12).

Electroacupuncture exerts neuroprotective effects through multiple pathways. It has been shown to upregulate brain-derived neurotrophic factor (BDNF) and nerve growth factor (NGF), promoting neuronal survival and axonal regeneration (15). Additionally, electroacupuncture inhibits neuroinflammation by suppressing the NF-κB/NLRP3 signaling pathway and reducing pro-inflammatory cytokines including TNF-*α*, IL-1β, and IL-6 (32, 33), Recent evidence suggests that electroacupuncture at 2/100 Hz alternating frequency activates both mu-opioid and delta-opioid receptor systems, optimizing the neuromodulatory response (34).

Body acupuncture at selected acupoints (Baihui, Neiguan, Zusanli) has been demonstrated to improve cerebral blood perfusion, regulate autonomic nervous system balance, and modulate the gut-brain axis (16). The TCM concept of “treating phlegm and blood stasis” underlying our acupoint selection corresponds to modern understanding of microcirculatory improvement and anti-inflammatory effects (35). Notably, the semi-standardized body acupuncture approach used in this study, with individualized point selection based on syndrome differentiation, may have provided more targeted systemic regulation compared with a fully standardized protocol, potentially enhancing treatment responsiveness.

The combined protocol likely produces synergistic effects by simultaneously targeting central neuroplasticity (scalp acupuncture), peripheral neuromuscular activation (electroacupuncture), and systemic regulation (body acupuncture), thereby creating a multi-level, multi-target therapeutic environment that may optimize the rehabilitation milieu for motor recovery. However, it should be acknowledged that the present study did not include biomarkers of neuroplasticity (e.g., serum BDNF levels, functional MRI measures), and the mechanistic explanations offered here remain speculative and require direct investigation in future studies.

### 4.3 Clinical implications

Our findings have several important clinical implications. First, the magnitude of improvement observed (9.13-point FMA difference) translates to meaningful functional gains for patients, potentially enabling progression from dependent to assisted or independent mobility (36). Second, the favorable safety profile supports the feasibility of implementing combined acupuncture protocols in clinical practice. Third, the sustained benefits at 12-week follow-up suggest that acupuncture-induced improvements are not merely transient but contribute to lasting functional recovery.

The multivariate regression analysis identified several predictors of treatment response, including younger age, lower baseline severity, and shorter time from onset. These findings suggest that early initiation of combined acupuncture therapy in the subacute phase may optimize therapeutic outcomes, consistent with the concept of capitalizing on the “critical window” of neuroplasticity (37).

From a health economics perspective, improved functional outcomes and quality of life may translate to reduced long-term healthcare utilization and caregiver burden, though formal cost-effectiveness analysis was beyond the scope of this study.

### 4.4 Strengths and limitations

This study has several strengths. First, the comprehensive combined acupuncture protocol reflects contemporary clinical practice and incorporates evidence-based acupoint selection. Second, multiple validated outcome measures captured both impairment-level (FMA, NIHSS) and participation-level (MBI, SS-QOL, mRS) outcomes. Third, propensity score matching analysis addressed potential selection bias, and results remained robust. Fourth, the 12-week follow-up period allowed assessment of sustained treatment effects.

Several limitations should be acknowledged. First, the retrospective design precludes causal inference, and residual confounding from unmeasured variables -such as differences in patient motivation, family support, socioeconomic status, and subtle variations in rehabilitation intensity- cannot be excluded despite propensity score matching. Second, although outcome assessors were blinded to treatment allocation, the lack of a sham acupuncture control group means that non-specific effects, including placebo responses, therapeutic attention bias, and patient expectation effects, may have contributed to the observed between-group differences. These psychological factors are particularly relevant for semi-subjective measures such as the SS-QOL and certain FMA components. Third, the single-center design and restriction to patients aged 40–75 with moderate stroke severity (NIHSS 5–15) limit the generalizability of findings to other populations, including younger patients, those with mild or severe strokes, and those in different healthcare settings. Fourth, objective biomarkers of neuroplasticity—such as serum BDNF and NGF levels, functional MRI connectivity measures, and diffusion tensor imaging metrics—were not evaluated, precluding direct assessment of the neurobiological mechanisms underlying clinical improvement. Fifth, the 12-week follow-up period, while demonstrating sustained benefits, is relatively short for evaluating long-term functional outcomes and the durability of treatment effects. Sixth, the semi-standardized body acupuncture protocol, while reflecting real-world practice, introduces additional variability that may affect reproducibility across different clinical settings. Seventh, as a multicomponent intervention, the present design does not allow determination of the individual contributions of scalp acupuncture, electroacupuncture, or body acupuncture to the observed effects.

### 4.5 Future directions

Future research should address several key gaps identified in this study. First, large-scale, multicenter randomized controlled trials with sham acupuncture controls are needed to establish definitive efficacy and to disentangle specific acupuncture effects from non-specific placebo responses. Sham acupuncture designs using non-penetrating needles at non-acupoints or minimal acupuncture at irrelevant sites would be particularly informative. Second, dismantling study designs using factorial analysis should be employed to determine the relative contribution and potential synergistic effects of each acupuncture modality (scalp acupuncture, electroacupuncture, and body acupuncture), thereby identifying the optimal combination for clinical practice. Third, incorporation of objective neuroimaging biomarkers (functional MRI, diffusion tensor imaging) and serum biomarkers (BDNF, NGF, inflammatory cytokines) would elucidate the neurobiological mechanisms underlying clinical improvement and help identify predictive biomarkers of treatment response, enabling personalized treatment selection. Fourth, investigation of optimal treatment timing (acute vs. subacute vs. chronic phase), duration (4 weeks vs. 8 weeks), and frequency (daily vs. alternate days) could inform protocol optimization for different patient profiles. Fifth, longer-term follow-up exceeding 1 year is essential to determine whether the observed benefits are durable or diminish over time. Finally, formal cost-effectiveness analysis incorporating direct medical costs, indirect costs (caregiver burden, lost productivity), and quality-adjusted life years would provide critical information for healthcare policy decisions and reimbursement considerations.

## 5 Conclusion

This retrospective cohort study demonstrates that combined acupuncture therapy (scalp acupuncture, electroacupuncture, and body acupuncture) integrated with conventional rehabilitation is associated with significantly improved motor function recovery and quality of life in patients with subacute stroke compared with conventional rehabilitation alone. The acupuncture group achieved significantly greater improvements in Fugl-Meyer Assessment scores (mean difference 9.13 points), Modified Barthel Index, NIHSS scores, and Stroke-Specific Quality of Life Scale, with benefits sustained at 12-week follow-up. Favorable functional outcome (mRS 0–2) was three times more likely in the acupuncture group. The treatment demonstrated an excellent safety profile. These findings support the potential integration of combined acupuncture therapy into comprehensive post-stroke rehabilitation programs. However, as the associations observed in this retrospective study may be influenced by unmeasured confounders and non-specific treatment effects, confirmation through rigorous multicenter randomized controlled trials with sham acupuncture controls is warranted.

## Data Availability

The raw data supporting the conclusions of this article will be made available by the authors, without undue reservation.

## References

[1] Feigin VL Brainin M Norrving B Martins SO Pandian J Lindsay P . World stroke organization: global stroke fact sheet 2025. Int J Stroke. (2025) 20:132–44. doi: 10.1177/17474930241308142, 39635884 PMC11786524

[2] Campbell BC. Hyperacute ischemic stroke care-current treatment and future directions. Int J Stroke. (2024) 19:718–26. doi: 10.1177/17474930241267353, 39096172 PMC11298121

[3] Liu T Li X Zhou X Chen W Wen A Liu M . PI3K/AKT signaling and neuroprotection in ischemic stroke: molecular mechanisms and therapeutic perspectives. Neural Regen Res. (2025) 20:2758–75. doi: 10.4103/NRR.NRR-D-24-00568, 39435629 PMC11826468

[4] Winstein CJ Stein J Arena R Bates B Cherney LR Cramer SC . Guidelines for adult stroke rehabilitation and recovery: a guideline for healthcare professionals from the American Heart Association/American Stroke Association. Stroke. (2016) 47:e98–e169. doi: 10.1161/STR.0000000000000098, 27145936

[5] Rajashekar D Boyer A Larkin-Kaiser KA Dukelow SP. Technological advances in stroke rehabilitation: robotics and virtual reality. Phys Med Rehabil Clin N Am. (2024) 35:383–98. doi: 10.1016/j.pmr.2023.06.026, 38514225

[6] Zhang Y Tang YW Peng YT Yan Z Zhou J Yue ZH. Acupuncture, an effective treatment for post-stroke neurologic dysfunction. Brain Res Bull. (2024) 215:111035. doi: 10.1016/j.brainresbull.2024.111035, 39069104

[7] Choi TY Jun JH Lee HW Yun JM Joo MC Lee MS. Traditional Chinese medicine interventions in the rehabilitation of cognitive and motor function in patients with stroke: an overview and evidence map. Front Neurol. (2022) 13:885095. doi: 10.3389/fneur.2022.885095, 35655620 PMC9152210

[8] Qin S Zhang Z Zhao Y Liu J Qiu J Gong Y . The impact of acupuncture on neuroplasticity after ischemic stroke: a literature review and perspectives. Front Cell Neurosci. (2022) 16:817732. doi: 10.3389/fncel.2022.817732, 36439200 PMC9685811

[9] Rao R Gan L Zhao R Han Y. Electroacupuncture alleviates cerebral ischemia injury by regulating PI3K/AKT/NF-κB signaling in microglia of ischemic stroke rats. Neuroreport. (2025) 36:22–30. doi: 10.1097/WNR.0000000000002115, 39651717

[10] Sun X Zhang A Pang B Wu Y Shi J Zhang N . Electroacupuncture pretreatment alleviates spasticity after stroke in rats by inducing the NF-κB/NLRP3 signaling pathway and the gut-brain axis. Brain Res. (2024) 1822:148643. doi: 10.1016/j.brainres.2023.148643, 37884180

[11] Tian Y Zhao P Peng Y. Effectiveness of scalp acupuncture therapy combined with training on limb movement disorders after stroke: a systematic review and meta-analysis. Eur J Integr Med. (2025) 74:102432. doi: 10.1016/j.eujim.2025.102432

[12] Zhang C Pang T Chen Y Lai P Nie R Wang Y . Interactive dynamic scalp acupuncture enhances brain functional connectivity in bilateral basal ganglia ischemic stroke patients: a randomized controlled trial. Front Neurol. (2025) 16:1604342. doi: 10.3389/fneur.2025.1604342, 40881782 PMC12381643

[13] Zhao M. Shen H. Yan X. Gao X. Liu Y. Zhou S. . Electroacupuncture modulates multiple pathways for neuroprotection and neurorepair in ischemic stroke. CNS Neurosci Ther. (2026) 32:e70862. doi: 10.1002/cns.70862 41944596 PMC13055200

[14] Xie H Gao Z Fan Y Shi J Tang Y Cha B . Clinical observation of acupuncture combined with modern rehabilitation in the treatment of limb motor dysfunction after ischemic stroke: a randomized controlled trial. Medicine (Baltimore). (2022) 101:e31703. doi: 10.1097/md.0000000000031703, 36397362 PMC9666196

[15] Liu SD Ye T Shen JQ . The effect of repetitive transcranial magnetic stimulation combined with electroacupuncture on upper limb motor function and serum BDNF, NGF in patients with stroke hemiplegia during recovery period. Chin J Gerontol. (2023) 43:2578–81. doi: 10.3969/j.issn.1005-9202.2023.11.005

[16] Zhang SH Wang YL Zhang CX Zhang CP Xiao P Li QF . Effects of interactive dynamic scalp acupuncture on motor function and gait of lower limbs after stroke: a multicenter, randomized, controlled clinical trial. Chin J Integr Med. (2022) 28:483–91. doi: 10.1007/s11655-021-3525-0, 34913147

[17] Ke C Zhou Z Sun M Shi W Xu M Su R . Acupuncture and stroke motor rehabilitation: a decade of evidence synthesis via systematic mapping (2015-2024). Front Neurol. (2025) 16:1647086. doi: 10.3389/fneur.2025.1647086, 41079346 PMC12511885

[18] Fugl-Meyer AR Jääskö L Leyman I Olsson S Steglind S. The post-stroke hemiplegic patient. 1. A method for evaluation of physical performance. Scand J Rehabil Med. (1975) 7:13–31. doi: 10.2340/1650197771331, 1135616

[19] Williams LS Weinberger M Harris LE Clark DO Biller J. Development of a stroke-specific quality of life scale. Stroke. (1999) 30:1362–9. doi: 10.1161/01.str.30.7.1362, 10390308

[20] Shah S Vanclay F Cooper B. Improving the sensitivity of the Barthel index for stroke rehabilitation. J Clin Epidemiol. (1989) 42:703–9. doi: 10.1016/0895-4356(89)90065-6, 2760661

[21] Brott T Adams HP Jr Olinger CP Marler JR Barsan WG Biller J . Measurements of acute cerebral infarction: a clinical examination scale. Stroke. (1989) 20:864–70. doi: 10.1161/01.str.20.7.864 2749846

[22] van Swieten JC Koudstaal PJ Visser MC Schouten HJ van Gijn J. Interobserver agreement for the assessment of handicap in stroke patients. Stroke. (1988) 19:604–7. doi: 10.1161/01.STR.19.5.604, 3363593

[23] Lin KC Fu T Wu CY Hsieh CJ. Assessing the stroke-specific quality of life for outcome measurement in stroke rehabilitation: minimal detectable change and clinically important difference. Health Qual Life Outcomes. (2011) 9:5. doi: 10.1186/1477-7525-9-5, 21247433 PMC3034658

[24] Liu J Meng X He Y Jiang H Zhang J Shi J . Clinical efficacy of electroacupuncture antagonistic muscles combined with rehabilitation training in the treatment of spastic hemiplegia after stroke: a systematic review and meta-analysis of randomized controlled trials. Front Neurol. (2025) 16:1634845. doi: 10.3389/fneur.2025.1634845, 40860970 PMC12370752

[25] Li L Zhu W Lin G Chen C Tang D Lin S . Effects of acupuncture in ischemic stroke rehabilitation: a randomized controlled trial. Front Neurol. (2022) 13:897078. doi: 10.3389/fneur.2022.897078, 35812118 PMC9260687

[26] Hou Z Gao S Wang F Wu J. Electroacupuncture combined with wet compress formula ameliorates upper limb dysfunction associated with stroke: a retrospective analysis of 126 cases. J Stroke Cerebrovasc Dis. (2024) 33:107524. doi: 10.1016/j.jstrokecerebrovasdis.2023.107524, 38103448

[27] Zhan Y Pei J Wang J Fu Q Xu J Yan M . Motor function and fALFF modulation in convalescent-period ischemic stroke patients after scalp acupuncture therapy: a multi-centre randomized controlled trial. Acupunct Med. (2023) 41:86–95. doi: 10.1177/09645284221086289, 35673804

[28] Jianfei S Zhengyuan Q Xinlu GU Yan Z Xingrui LI. Efficacy of acupuncture combined with upper limb rehabilitation robot-assisted training for neuroplasticity and functional recovery of patients with stroke: a prospective cohort study based on functional near-infrared spectroscopy technology. J Tradit Chin Med. (2025) 45:860–6. doi: 10.19852/j.cnki.jtcm.2025.04.015, 40810232 PMC12340582

[29] Lin D Gao J Lu M Han X Tan Z Zou Y . Scalp acupuncture regulates functional connectivity of cerebral hemispheres in patients with hemiplegia after stroke. Front Neurol. (2023) 14:1083066. doi: 10.3389/fneur.2023.1083066, 37305743 PMC10248137

[30] Zhang J Lu C Wu X Nie D Yu H. Neuroplasticity of acupuncture for stroke: an evidence-based review of MRI. Neural Plast. (2021) 2021:2662585. doi: 10.1155/2021/2662585 34456996 PMC8397547

[31] Zhang Y Lu H Ren X Zhang J Wang Y Zhang C . Immediate and long-term brain activation of acupuncture on ischemic stroke patients: an ALE meta-analysis of fMRI studies. Front Neurosci. (2024) 18:1392002. doi: 10.3389/fnins.2024.1392002, 39099634 PMC11294246

[32] Chavez LM Huang SS MacDonald I Lin JG Lee YC Chen YH. Mechanisms of acupuncture therapy in ischemic stroke rehabilitation: a literature review of basic studies. Int J Mol Sci. (2017) 18:2270. doi: 10.3390/ijms18112270, 29143805 PMC5713240

[33] Dai M Zhao Y Jia Z Xu S Xu N Wu X . Effect of specific mode electroacupuncture stimulation combined with NGF during the ischaemic stroke: study protocol for a randomized controlled trial. Clinics (Sao Paulo). (2024) 79:100451. doi: 10.1016/j.clinsp.2024.100451, 39033586 PMC11325668

[34] Chen L Fang J Ma R Gu X Chen L Li J . Additional effects of acupuncture on early comprehensive rehabilitation in patients with mild to moderate acute ischemic stroke: a multicenter randomized controlled trial. BMC Complement Altern Med. (2016) 16:226. doi: 10.1186/s12906-016-1193-y, 27430340 PMC4950630

[35] Chinese Society of Neurology. Chinese guidelines for diagnosis and treatment of acute ischemic stroke 2023. Chin J Neurol. (2024) 57:523–59.

[36] Kwakkel G Lannin NA Borschmann K English C Ali M Churilov L . Standardized measurement of sensorimotor recovery in stroke trials: consensus-based Core recommendations from the stroke recovery and rehabilitation roundtable. Neurorehabil Neural Repair. (2017) 31:784–92. doi: 10.1177/1545968317732662, 28934918

[37] Joy MT Carmichael ST. Encouraging an excitable brain state: mechanisms of brain repair in stroke. Nat Rev Neurosci. (2021) 22, 38–53. doi: 10.1038/s41583-020-00396-7 33184469 PMC10625167

