# Supplementary material for: Acupuncture for post-stroke recovery: a retrospective cohort study on motor function and quality of life
Source: Front Neurol. 2026 May 4;17:1794260. doi: 10.3389/fneur.2026.1794260 (PMC13181924; doi:10.3389/fneur.2026.1794260)
Supplement: Supplementary file 1 [file Table_1.docx]

**Supplementary Table S1. Propensity Score Matching Analysis – Covariate Balance**

| **Variable** | **Before SMD** | **Before P** | **After SMD** | **After P** |
| --- | --- | --- | --- | --- |
| Age | 0.162 | 0.412 | 0.048 | 0.826 |
| Sex (Male) | 0.039 | 0.843 | 0.021 | 0.918 |
| Stroke type (Ischemic) | 0.054 | 0.782 | 0.028 | 0.892 |
| Onset to treatment (days) | 0.208 | 0.278 | 0.065 | 0.752 |
| Baseline NIHSS | 0.138 | 0.472 | 0.042 | 0.838 |
| Hypertension | 0.043 | 0.827 | 0.025 | 0.904 |
| Diabetes mellitus | 0.039 | 0.839 | 0.018 | 0.932 |

*Abbreviations: PSM, propensity score matching; SMD, standardized mean difference; NIHSS, National Institutes of Health Stroke Scale.*

*SMD<0.1 indicates adequate covariate balance. After PSM: n=48 per group.*
